# *Chlamydia muridarum* mutant CM-pGP3S as a novel attenuated live rectal vaccine protects against genital tract infection

**DOI:** 10.3389/fcimb.2025.1550455

**Published:** 2025-05-13

**Authors:** Jingyue Ma, Tianyuan Zhang, Qi Tian, Meng Xiao, Long Han, Quanzhong Liu, Guangming Zhong, Yuanjun Liu

**Affiliations:** ^1^ Department of Dermatovenereology, Tianjin Medical University General Hospital/Tianjin Institute of Sexually Transmitted Disease, Tianjin, China; ^2^ Shanghai Institute of Virology, Shanghai Jiao Tong University School of Medicine, Shanghai, China; ^3^ Department of Obstetrics and Gynecology, Hunan Provincial Maternal and Child Health Care Hospital, Changsha, Hunan, China; ^4^ Department of Microbiology, Immunology and Molecular Genetics, University of Texas Health Science Center at San Antonio, Texas, TX, United States

**Keywords:** *Chlamydia muridarum*, attenuated rectal vaccine, pGP3S, mucosal immunity, rectal immunization, genital tract infection

## Abstract

**Introduction:**

*Chlamydia trachomatis* (CT) is a major sexually transmitted pathogen with severe complications. Using *Chlamydia muridarum* (CM) as a model, this study evaluates the attenuated mutant *Chlamydia muridarum* (CM-pGP3S) as a novel rectal vaccine to protect against genital tract infection and pathology.

**Methods:**

Female C57BL/6 mice were rectally immunized with low (1×10^3^), middle (1×10^5^), or high (1×10^7^) doses of CM-pGP3S. Mice were challenged intravaginally with wild-type *Chlamydia muridarum* 63 days post-immunization. Protection was assessed via genital *Chlamydia* shedding, hydrosalpinx incidence (gross/histopathology), serum IgG, fecal IgA, and T cell responses. Gut microbiota stability was analyzed using qPCR.

**Results:**

CM-pGP3S immunization significantly reduced CM-WT genital shedding duration (3–7 days vs. 21 days in controls, *p* < 0.01) and hydrosalpinx incidence (0% vs. 80% in controls, *p* < 0.01). Elevated systemic and mucosal immunity were observed, including higher serum IgG (1:100–1:1600 dilutions, *p* < 0.05–0.01) and fecal IgA (*p* < 0.05–0.01). CD4^+^ and CD8^+^ T cells exhibited increased IFN-γ (*p* < 0.01), while CD8^+^ T cells showed elevated TNF-α and IL-2 (*p* < 0.05). No colitis or significant gut microbiota disruption occurred post-immunization.

**Discussion:**

Rectal CM-pGP3S vaccination induces robust transmucosal immunity, protecting against genital *Chlamydia *infection and pathology without gastrointestinal adverse effects. This highlights its potential as a safe and effective mucosal vaccine strategy to combat CT genital tract infections.

## 1 Introduction

*Chlamydia trachomatis* (CT) genital infection is a prevalent sexually transmitted disease globally (Workowski et al., 2021). A systematic review of 100 studies estimated CT’s global prevalence at approximately 3.8% among women aged 15 to 49 years in 2016 (Hu et al., 2021). The clinical symptoms of CT genital infection are often mild and insidious, with 70-95% of men and 45-96% of women being asymptomatic (Dielissen et al., 2013). Chronic CT infection, however, can lead to severe complications, including epididymitis, prostatitis, and proctitis in men, and cervicitis, urethritis, pelvic inflammatory disease, and ectopic pregnancy in women. In severe cases, it can result in infertility (Mohseni et al., 2025). Treatment failures are common due to factors such as inappropriate dosage, improper drug selection, and insufficient treatment duration, complicating CT management (Kong and Hocking, 2015). Although much progress has been made in understanding CT infection, developing an effective vaccine remains one of the most promising approaches for CT prevention and control (Poston et al., 2019).

Research on *Chlamydia* vaccines has gained attention since the 1950s, leading to the development of Deoxyribonucleic acid (DNA), subunit, and live attenuated vaccines (McIntosh, 2020). Vaccine delivery systems, novel adjuvants, and delivery methods have also played key auxiliary roles in *Chlamydia* vaccine research (Borges et al., 2022). A protective vaccine targeting the major outer membrane protein of *Chlamydia* has entered early clinical trials, but no *Chlamydia* vaccine has yet been approved for human use, making further research essential (de la Maza et al., 2021). Previous vaccines have primarily used vaginal or intramuscular administration, which can cause discomfort and inconvenience for patients and healthcare providers. Mucosal immunization, which leverages the common mucosal immune system to elicit immune responses at various mucosal sites, presents a practical alternative. This approach can stimulate Immunoglobulin (Ig) A production to block pathogen invasion (Li et al., 2020). The intestinal mucosa, a primary site for foreign pathogen entry, is particularly suited for vaccine delivery (Azegami et al., 2014). This method is straightforward and safe, bypassing stringent vaccination standards and facilitating implementation (Li et al., 2020). Since CT is often detected in the digestive tract after genital tract infection in females (Zhong, 2018; Dirks et al., 2019), gastrointestinal vaccination against *Chlamydia* may represent a convenient and effective new transmucosal vaccination strategy (Morrison et al., 2020).

While the medical significance of CT in the human digestive tract remains unclear, studies have shown that *Chlamydia muridarum* (CM) can survive and colonize the mouse digestive tract, thereby reducing genital CM infection and upper genital tract pathology (Wang et al., 2018; Zhong, 2018). CM causes urogenital infections in mice, mirroring human CT infections, making the CM mouse genital tract model widely used for studying CT pathogenesis and immune protection (Lundy et al., 2021). Previous research has demonstrated that administering CM-wild-type (CM-WT) orally in the upper digestive tract can provide substantial transmucosal protection, reducing the severity and duration of CM genital infection and related hydrosalpinx pathology (Wang et al., 2018). Colonization of CM-WT in the upper digestive tract did not result in significant intestinal pathology, noticeable epithelial inflammation, or major shifts in gut flora (Yeruva et al., 2013; Ma et al., 2020). To enhance vaccine safety, an attenuated CM strain lacking plasmid-encoded Glycoprotein 3 (with a premature stop codon in the pgp3 gene, termed CM-pGP3S) has been evaluated in mice (Hou et al., 2018). Oral administration of CM-pGP3S successfully induced robust transmucosal immunity against CM-WT genital infection and pathogenicity (Zhou et al., 2022).

However, it remains uncertain whether oral administration is the optimal route. Rectal administration may offer superior protection. Several reasons could account for rectal immunization being superior to oral immunization. Oral vaccines may be inactivated by gastric acid or digestive enzymes, and PGP3S-CM cannot be found in the digestive tract when degraded (Zhang et al., 2019), so the immune effect may be poor. Rectal administration can bypass the degradation of the upper gastrointestinal tract and improve the stability of the vaccine. Two studies showed the results of a medium-dose oral vaccine (Wang et al., 2018; Zhou et al., 2022), but the present study investigated whether a low-dose CM-pGP3S rectal vaccine could induce a strong immune protection effect in the vagina. Furthermore, it has been reported that humoral and cellular immunity can be induced by rectal inoculation (Pais et al., 2017; Varotto-Boccazzi et al., 2023).

This study explores whether rectal inoculation with the CM-pGP3S vaccine could generate transmucosal immune responses that protect mice against subsequent CM-WT genital infection and associated pathology. Our findings suggest that CM-pGP3S may be developed into a safe rectal vaccine to induce protective immunity in the female genital tract.

## 2 Materials and methods

### 2.1 Animals

All animals were housed in a pathogen-free environment and provided food ad libitum. A total of 80 six-week-old female C57BL/6 mice obtained from Beijing Charles River Laboratories were used in this study. Although it is known that this strain of mice may clear infection faster and show a lower baseline pathological level, the model is identical to that of C57BL/6 mice in previous studies using CM oral vaccine (Wang et al., 2018; Zhou et al., 2022). The mice were maintained under specific pathogen-free (SPF) conditions at Tianjin Medical University General Hospital’s animal facility, with controlled temperature (22 ± 1°C) and relative humidity (55 ± 5%). They were allowed a 7-day acclimation period before the experiment.

All procedures for the care and use of animals were approved by the Ethics Committee of Tianjin Medical University General Hospital (IRB2022-DWFL-129), and all institutional and governmental regulations regarding the ethical use of animals were followed. All immunizations, challenges, and surgery were performed under 2% isoflurane, and all efforts were made to minimize suffering.

### 2.2 Cell culture

HeLa cells were provided by the Tianjin Institute of Sexually Transmitted Diseases. CM and rabbit anti-CM serum antibodies were kindly donated by Professor Guangming Zhong from the Microbial Immunology Department of the University of Texas Health Medical Center in San Antonio.

### 2.3 *Chlamydia* organism vaccines

The vaccine candidate used in this investigation consisted of CM with a premature stop codon in the pGP3 gene to create CM-pGP3S, as previously detailed (Hou et al., 2018). CM strains, including CM-WT and CM-pGP3S, were generously provided by Dr. Guangming Zhong’s lab at the University of Texas Health Science Center in San Antonio, USA. These strains had been previously titrated on HeLa cell monolayers, purified via renografin gradients, and stored at -80°C. All aliquots of the purified Elementary Bodies (EBs) were stored at -80°C until use.

### 2.4 Mice rectal immunization and vaginal challenge infection

Purified CM EBs were used to inoculate 6-week-old female C57BL/6J mice (Beijing Charles River Laboratories) rectally (as the immunization route) or intravaginally (as the challenge infection route) as previously described (Wang et al., 2018; Zhou et al., 2022). The EBs and RBs of *Chlamydia* were purified from cell cultures by density gradient centrifugation using Gastrografin^®^ and K-36 buffer. The steps involved centrifuging a partially purified EBs/RBs sample in a 20% Gastrografin gradient, followed by further separation in a 34%, 44%, and 54% Gastrografin discontinuous gradient, and finally collecting an EBs-enriched fraction from the 44%/54% interface (Klasinc et al., 2021). Then, 50 μL of the coated solution containing 1×10^5^ inclusion forming units (IFUs) CM-EBs was added to each well of the ELISA plate. Live CM-EBs were used for packet encapsulation. Three doses of CM-pGP3S (1×10³, 1×10^5^, and 1×10^7^ IFUs) were administered rectally. Subsequently, mice were challenged intravaginally with 2×10^5^ IFUs of CM-WT to assess CM-pGP3S protection, in line with prior studies (Wang et al., 2018; Zhou et al., 2022). To evaluate long-term protection, an intravaginal challenge was conducted 63 days post-rectal CM-pGP3S inoculation. The mice’s health status and infection levels were monitored by enumerating *Chlamydia* IFUs from vaginal and/or rectal swabs using indirect immunofluorescence. The mean number of IFUs at each time point was calculated. At the end of the experiment, mice were deeply anesthetized, and blood, feces, and the entire genital tract were collected for further research.

### 2.5 Titration of live *Chlamydia* organisms

To quantify live *Chlamydia* organisms in vaginal or rectal swabs, each swab was soaked in 0.5 mL of sucrose-phosphate-glutamate (SPG) buffer, vortexed with glass beads, and the released *Chlamydia* organisms in the supernatant were titrated on HeLa cell monolayers in duplicate. The infected cultures were processed for immunofluorescence assay as described (Liu et al., 2014). Inclusions were counted in five random fields per coverslip under a fluorescence microscope. For coverslips with fewer than one IFU per field, the entire coverslip was counted. The total number of IFUs per swab was calculated based on the mean IFUs per view, the ratio of the view area to that of the well, the dilution factor, and inoculation volumes. When possible, a mean IFUs/swab value was derived from serially diluted duplicate samples for each swab. The total IFUs/swab count was converted to log_10_, which was used to calculate the mean and standard deviation across mice of the same group at each time point.

### 2.6 Immunofluorescence assay

Briefly, infected HeLa cells grown on coverslips were fixed with 4% paraformaldehyde for 30 minutes at room temperature, followed by permeabilization with 2% (wt/vol) saponin (Sigma) for 60 minutes, then blocking. A 1:2000 dilution of rabbit anti-CM serum antibody was added and incubated at 37°C for 1 hour. Next, a 1:200 dilution of secondary fluorescent Fluorescein Isothiocyanate (FITC)-labeled goat anti-rabbit IgG antibody and a 1:1000 dilution of Hoechst blue fluorescent dye were added. Rabbit antibodies against CM, along with a secondary antibody conjugated with Cyanine Dyes (Cy) 2 (green; Abnova), were used to mark *Chlamydia* inclusions. Immunofluorescence images were obtained using a confocal laser scanning microscope (Carl Zeiss Microscopy GmbH-Axio Imager A2, Germany).

### 2.7 Gross and histopathological analysis of the genital tract after CM-pGP3S rectal inoculation

After the CM-WT intravaginal challenge, mice were euthanized to assess genital tract pathology, focusing on upper genital tract hydrosalpinx. Prior to removing the genital tract tissues, a gross examination was performed to detect oviduct hydrosalpinx or related abnormalities. The genital tract tissues were isolated, placed on a blue background, and imaged. Oviduct hydrosalpinx was visually scored based on dilation size using a scoring system as previously described (Conrad et al., 2016). Mice with hydrosalpinx on either side of the oviducts were classified as hydrosalpinx positive, and the severity of hydrosalpinx was scored as follows (Conrad et al., 2016): 0, no oviduct dilation (no hydrosalpinx); 1, hydrosalpinx visible only under a stereoscope; 2, hydrosalpinx visible to the naked eye but smaller than the ovary; 3, hydrosalpinx equal to the ovary in size; 4, hydrosalpinx larger than the ovary. Scores from both oviducts in each mouse were combined as the total pathology score. Hydrosalpinx incidence and severity scores were statistically analyzed between mice infected with CM-WT. Hydrosalpinx incidence was calculated as the number of mice with a bilateral score of 1 or higher divided by the total number of mice in the group.

### 2.8 Hematoxylin and eosin staining

For histological pathology observation, isolated genital tract tissues were fixed in 10% neutral formalin, embedded in paraffin, and sectioned longitudinally. Efforts were made to include the cervix, uterine horns, and oviducts, as well as luminal structures, in each section. The sections were stained with Hematoxylin and Eosin (H&E) as previously described (Conrad et al., 2016). H&E-stained sections were examined under a microscope for inflammation severity and pathology, following modified, established schemes. The severity of oviduct inflammatory infiltration was semi-quantitatively scored based on the following criteria (Conrad et al., 2016): 0, no significant inflammatory infiltration; 1, infiltration at one focus; 2, infiltration at two to four foci; 3, infiltration at more than four foci; 4, confluent infiltration. Images of each mouse’s oviduct dilation and inflammatory infiltration were taken under a 10X objective lens.

### 2.9 Gross and histopathological analysis of the lower digestive tract after CM-pGP3S rectal inoculation

On day 63 post-CM-pGP3S rectal immunization, mice in the high-dose group (1×10^7^) were euthanized to evaluate inflammation in the lower digestive tract. The tissues were isolated, laid on a blue background for imaging, and measured for length to assess potential shortening due to inflammation. Histopathological analysis of these tissues was conducted as described above.

### 2.10 Quantitative PCR

Fecal samples were collected from female C57BL/6J mice on various days after rectal immunization, with or without CM-pGP3S. Baseline fecal samples were collected from all mice prior to inoculation and designated as day 0 samples. Samples were frozen in liquid nitrogen and stored at -80°C until DNA extraction. Total genomic DNA was extracted from each sample (150 mg) using a QIAamp DNA stool mini kit (Qiagen Inc., Germantown, MD) per the manufacturer’s instructions. The DNA purity was assessed by determining absorbance ratios at 260 nm/280 nm. Real-time quantitative Polymerase Chain Reaction (qPCR) using a SYBR green-based method with iTaq universal SYBR green super mix 500 (Bio-Rad) quantified bacterial 16S ribosomal Ribonucleic Acid (rRNA) genes. The primers used were identical to those in previous studies (Wang et al., 2018). PCR conditions were 95°C for 15 s and 60°C for 30 s, repeated for 45 cycles. The number of PCR cycles required for a given amplicon level was used to calculate the relative abundance of the targeted template.

### 2.11 Enzyme-linked immunosorbent assays

Levels of CM-specific antibodies (IgG and IgA) in sera and fecal samples collected 63 days post-high-dose CM-pGP3S rectal inoculation (1×10^7^) were measured by standard ELISA procedures (Wang et al., 2018). A 50 µL coating solution containing activated CM EBs (1×10^5^) in a 50 mM carbonate/bicarbonate buffer was added to each well of a 96-well ELISA plate, followed by overnight incubation at 4°C. Plates were washed five times with phosphate-buffered saline (PBS) containing 0.05% Tween-20 for 3 minutes each. Plates were blocked with PBS containing 5% skim milk and incubated at room temperature for 2 hours. Serum samples collected from the tail vein were serially diluted 1:100 in PBS and incubated at 4°C for 2 hours. Fecal samples were resuspended in PBS to a final concentration of 1 mg/µL, centrifuged at 13000 rpm for 5 minutes, and supernatants were applied to 96-well plates with or without 2-fold serial dilutions. Horseradish peroxidase-labeled goat anti-mouse IgG (1:500) and IgA (1:1000) antibodies (Dingguo Biotechnology Co.) were added and incubated at 37°C for 1 hour. Absorbance Optical Density (OD) at 405 nm was measured using the 2,2’-Azinobis-(3-ethylbenzthiazoline-6-sulfonic acid) diammonium salt (ABTS) substrate.

### 2.12 Flow cytometry

Flow cytometry was used to monitor changes in T-cell inflammatory factors in mice. Dendritic cells were exposed to CM overnight based on a previously described method (Lu and Zhong, 1999). Dendritic cells were mixed with CM at an infection ratio of 10:1 (MOI=10) to allow full contact of EBs with dendritic cells, and the mixed suspension was then added into 24-well plates and incubated overnight at 37°C without centrifugation. Because centrifugation may interfere with the natural phagocytosis function of dendritic cells or pathogen structure, direct mixing is closer to the process of antigen uptake under physiological conditions. A high MOI (e.g., MOI=10) ensured that the EBs were in full contact with the dendritic cells without the need for additional centrifugation steps. Spleen cells from CM-pGP3S (1×10^7^) rectally inoculated mice were collected and incubate with EBs-pulsed dendritic cells to be used to present CM antigens to T cells overnight. After overnight *in vitro* antigen re-stimulation (10–14 hours), the Golgi stop protein transport inhibitor was added to the culture at a final concentration of 25 µg/mL for 6 hours. To identify T cell subsets and detect intracellular inflammatory factors, lymphocytes cultured *in vitro* were blocked with rat anti-mouse Fc receptor (Cluster of Differentiation (CD) 16/CD32) antibodies. Cells were then labeled with anti-CD4 and anti-CD8 antibodies, followed by antibodies against Tumor Necrosis Factor (TNF) -α, Interferon (IFN) -γ, Interleukin (IL) -5, and IL-2. After incubation, cells stained with specific antibody combinations were counted using Laser-based Sorting and Research Instrument II (LSRII) flow cytometry (BD Bioscience), as previously described. Data analysis was conducted using FlowJo software (FlowJo, LLC).

### 2.13 Statistical analysis

All statistical analyses were performed using Statistical Package for the Social Sciences (SPSS) Statistics 27 software (IBM Corp., USA). One-way analysis of variance (ANOVA) was used for comparisons among three or more groups. Differences between the two groups were compared using an unpaired Student’s *t*-test. Differences were considered statistically significant at *p* < 0.05.

## 3 Results

### 3.1 Effect of vaginal infection of CM-WT and CM-pGP3S on genital tract pathology

**Figure 1:**
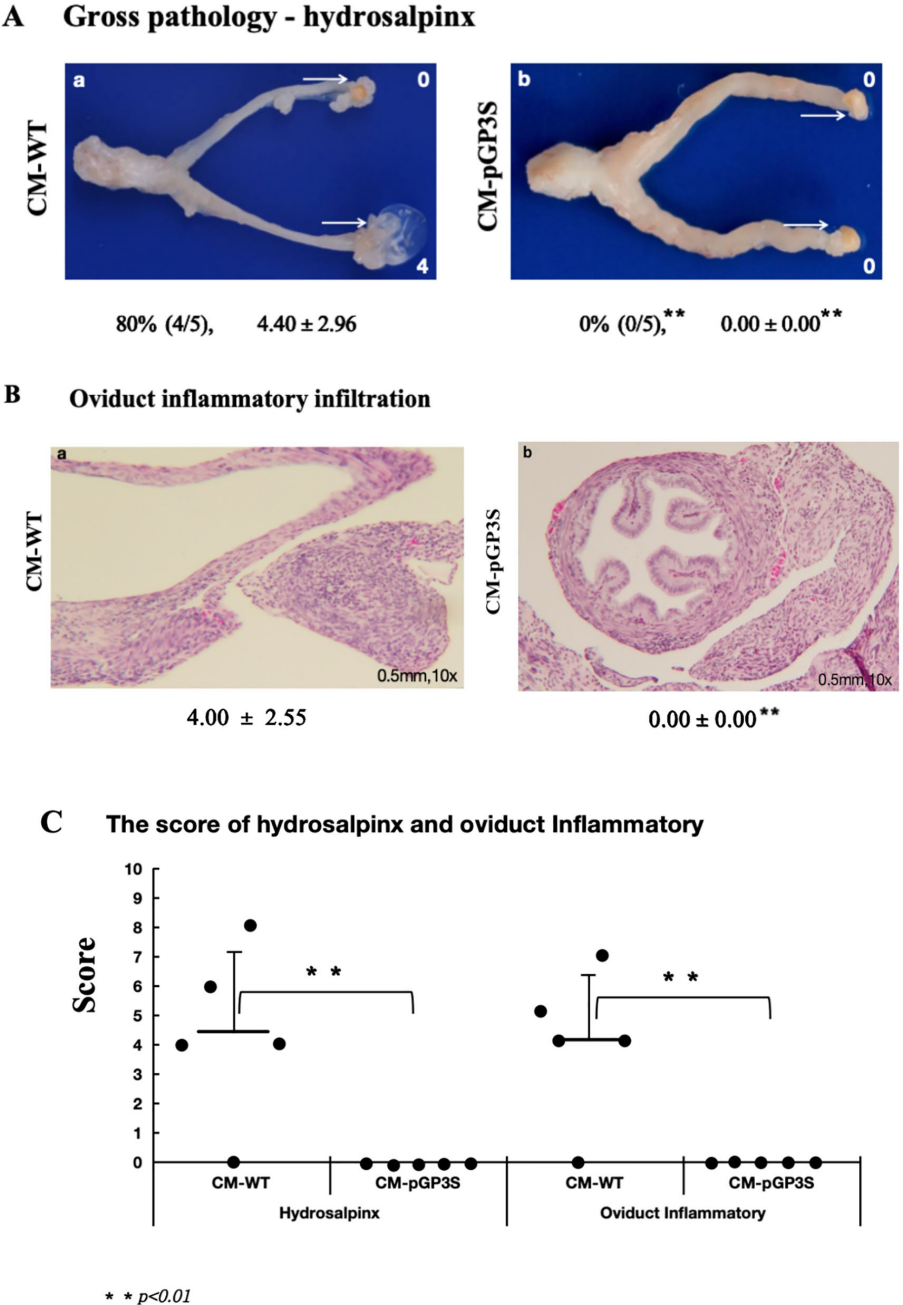
Effect of vaginal infection of CM-WT and CM-pGP3S on genital tract pathology. **(A, B)** C57BL/6J mice were infected vaginally with CM-WT (n=5) and CM-pGP3S (n=5) at 2×10^5 IFUs (Inclusion Forming Units). All mice were sacrificed after 63 days, and gross pathology and histopathological analyses of the genital tract were performed. **(C)** CM-WT induced significant hydrosalpinx and inflammatory changes in oviduct tissue under a 10x objective lens, while CM-pGP3S did not cause any pathological changes. The difference was statistically significant (**p<0.01).

Mice were infected vaginally with CM-WT and CM-pGP3S at 2×10^5^ IFUs. After 63 days, 80% of mice infected with CM-WT exhibited severe oviduct hydrosalpinx, with a gross tubal score of 4.40 ± 2.96. However, no hydrosalpinx was induced in mice infected with CM-pGP3S (*p* < 0.01) (**Figure 1A**). Tubal histopathology, verified by microscopy, showed severe chronic inflammatory cell infiltration in oviduct tissue of CM-WT-infected mice (inflammatory score 4.00 ± 2.55), while no inflammatory cell infiltration was evident in CM-pGP3S-treated mice (*p* < 0.01) (**Figure 1B**). There was a statistically significant difference in the scores of hydrosalpinx and oviduct inflammatory between the two group (p < 0.01) (**Figure 1C**). These findings align with the gross pathology results, indicating that CM-pGP3S caused no pathological changes in the genital tract compared to CM-WT.

### 3.2 CM-pGP3S rectal immunization induces transmucosal protection against vaginal challenge infection by CM-WT

**Figure 2:**
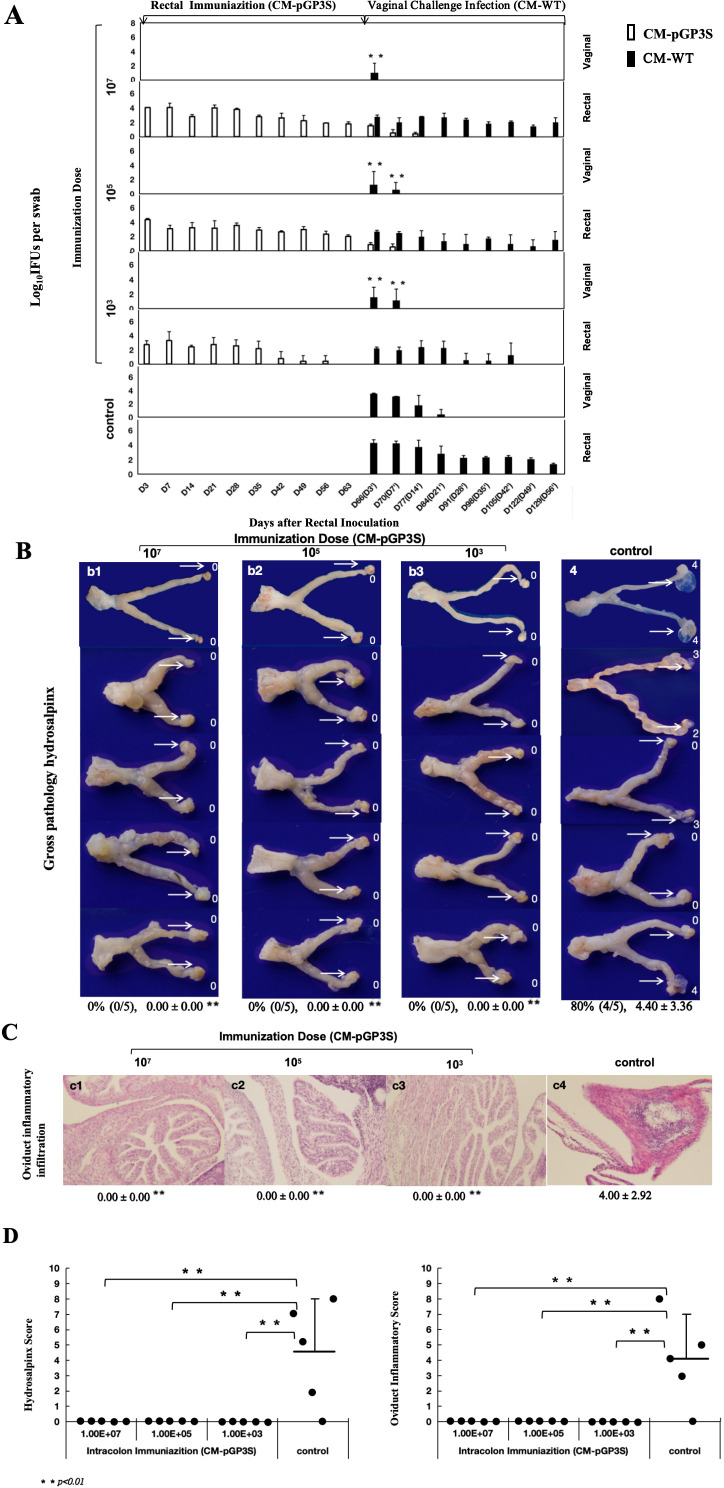
Effect of rectal immunization with attenuated CM-pGP3S vaccine on infectivity and pathogenicity against CM-WT vaginal challenge. **(A)** Four groups of C57BL/6J mice (n=5 per group) were monitored for live *Chlamydia* burdens in the genital tract. Vaginal and rectal swabs were collected on days 3, 7, and weekly thereafter following immunization and challenge infection. Results, expressed as Log10 IFUs per swab, showed a significant reduction in IFU shedding in CM-pGP3S-immunized mice compared to controls on days 3 and 7 (p<0.01). **(B)** Macroscopic and pathological images of the entire genital tract in both control and immunized groups were analyzed. Severely dilated oviducts, easily identified in the control group, had a hydrosalpinx incidence of 80% (4/5) with an average score of 4.40 ± 3.36 (a4). In contrast, CM-pGP3S-immunized mice at low (1×10^3, a1), middle (1×10^5, a2), and high doses (1×10^7, a3) did not develop hydrosalpinx following a 56-day CM-WT vaginal challenge (0/5). Histological examination with H&E staining under a 10x objective lens showed inflammatory infiltration in the oviduct lumen and tissue (b1-b4). Both hydrosalpinx and inflammatory scores were semi-quantitatively assessed as described in the materials and methods. The three immunized groups showed significantly reduced oviduct dilation (c1: **p<0.01) and inflammatory infiltration in the upper genital tract (c2: **p<0.01).

Rectal immunization with varying doses of CM-pGP3S in the gastrointestinal tract reduced CM-WT infection in the genital tract following vaginal challenge. CM-WT infections were monitored by the weekly collection of vaginal-cervical swabs from individual mice, with *Chlamydia* IFUs enumerated. Rectal immunization with low (1×10^3^), middle (1×10^5^), and high (1×10^7^) doses of CM-pGP3S significantly reduced the intensity and duration of CM-WT genital shedding (**Figure 2A**). Immunized mice challenged vaginally with CM-WT exhibited genital infection for only 3 to 7 days, compared to 21 days in the control group (non-immunized mice), with substantial reductions in genital shedding (*p* < 0.01). By day 14 post-challenge, CM-WT organisms were undetectable in immunized mice, whereas control mice exhibited persistent shedding until day 21 (**Figure 2A**).

Further evaluation of genital pathology was performed at both macroscopic (**Figure 2B-a**) and microscopic levels (**Figure 2C**). Vaginal challenge with CM-WT induced significant hydrosalpinx in 80% of the control mice, with a gross tubal score of 4.40 ± 3.36, whereas no hydrosalpinx cases were detected in the CM-pGP3S group. To validate these findings, genital tissues were stained with H&E, showing clear oviduct dilation and inflammatory infiltration in control mice with hydrosalpinx. The oviduct inflammatory score for the control group was 4.00 ± 2.92. In contrast, rectally immunized mice in the low, middle, and high-dose groups had no inflammatory score, indicating significant protection from oviduct hydrosalpinx and inflammatory infiltration induced by CM-WT vaginal challenge (**Figure 2D**).

### 3.3 The lower digestive tract immunized with CM-pGP3S is non-pathogenic

**Figure 3:**
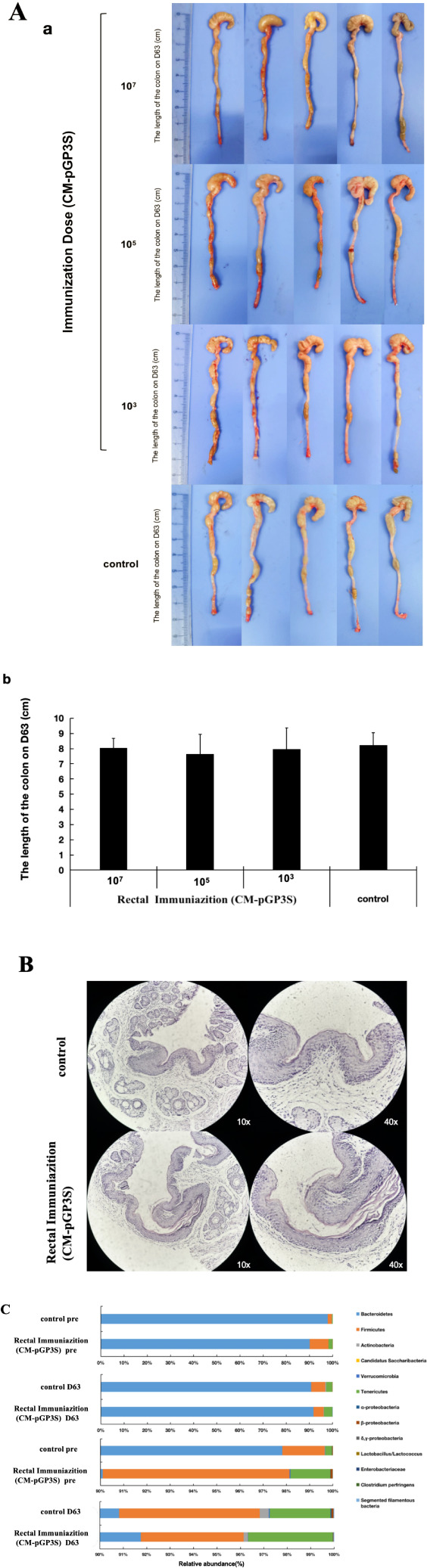
Effects of rectal immunization with attenuated CM-pGP3S vaccine on lower digestive tract. **(A)** Macroscopic images of the lower digestive tract in the three CM-pGP3S rectal immunization groups and the control group (n=5). No significant differences were observed in gross appearance **(a)** or colon length **(b)** across low (1×10^3), middle (1×10^5), high dose (1×10^7), and control groups on day 63. **(B)** Pathological biopsies of colonic epithelial tissue showed no significant differences between the high-dose group and controls after 63 days (n=5). **(C)** Analysis of intestinal flora in high-dose (n=5) and control (n=5) groups revealed stable increases in the phylum Tenericutes in all groups on day 63.

The impact of rectal CM-pGP3S vaccination on the digestive tract was evaluated through macroscopic observation, pathology, and gut microbiota analysis. After 63 days, the colons were examined carefully. No significant differences were observed in the gross appearance (**Figure 3A-a**) or length of the colon between mice that received *Chlamydia* rectal inoculation and control mice (**Figure 3A-b**, p > 0.05), indicating that CM-pGP3S did not cause colitis in the digestive tract.

Mice that received a high dose (1×10^7^) of CM-pGP3S underwent pathological examination, which showed that the epithelial tissue structure remained intact. Adjacent sections stained with H&E demonstrated no significant inflammatory infiltration in colon tissue between immunized and control mice (**Figure 3B**). We further assessed the effects of CM-pGP3S on intestinal flora (**Figure 3C**). Fecal samples from C57BL/6J mice that underwent high-dose CM-pGP3S rectal inoculation and control mice were analyzed by qPCR for seven major bacterial phyla, including four subclasses of the phylum *Proteobacteria*. Compared with the control group, the proportion of *Verrucomicrobia* and β-*Proteobacteria* in the CM-pGP3S inoculation group showed a slight decrease (**Table 1**, *p* < 0.05) on day 63. Upon closely examining the less abundant phyla, we found that the phylum *Tenericutes* in all groups showed a stable increase, irrespective of CM-pGP3S rectal immunization on day 63. These time-dependent changes in the intestinal flora were not significantly affected by lower digestive tract CM-pGP3S.

**Table 1:** Comparison of the proportion of intestinal flora between the two groups of mice.

Treatment group	Bacteroidetes	Firmicutes	Actino	Candidatus Sacchari	Verruco	Tenericutes	α-proteo	β-proteo	δ,γ-proteo	Lacto	Entero	Clostridium perfringens	Segmented filamentous bacteria
			bacteria	bacteria	microbia		bacteria	bacteria	bacteria	bacillus	bacteriaceae		
CM-pGP3S Immuniazition	9.17E-01±	4.39E-02±	1.83E-03±	1.21E-07±	1.38E-04±	3.61E-02±	9.22E-05±	2.65E-04±	7.42E-08±	1.61E-06±	6.31E-06±	2.27E-08±	2.48E-04±
D63	1.01E-01	8.43E-02	2.31E-03	1.01E-07	1.96E-04^*^	1.98E-02	1.74E-04	2.09E-04^*^	2.40E-08	6.69E-07	9.84E-06	2.73E-08	1.41E-04
Control	9.08E-01±	6.00E-02±	3.84E-03±	3.89E-07±	4.02E-04±	2.60E-02±	3.48E-04±	9.30E-04±	3.86E-07±	9.67E-07±	7.52E-06±	8.97E-08±	2.62E-04±
	1.02E-01	5.34E-02	1.41E-03	3.58E-07	1.29E-04^*^	4.80E-02	1.20E-04	2.73E-04^*^	4.14E-08	6.75E-07	3.58E-06	4.53E-08	1.72E-04

(*, p<0.05).

### 3.4 The humoral and cellular immunity of CM-pGP3S rectal immunization

**Figure 4:**
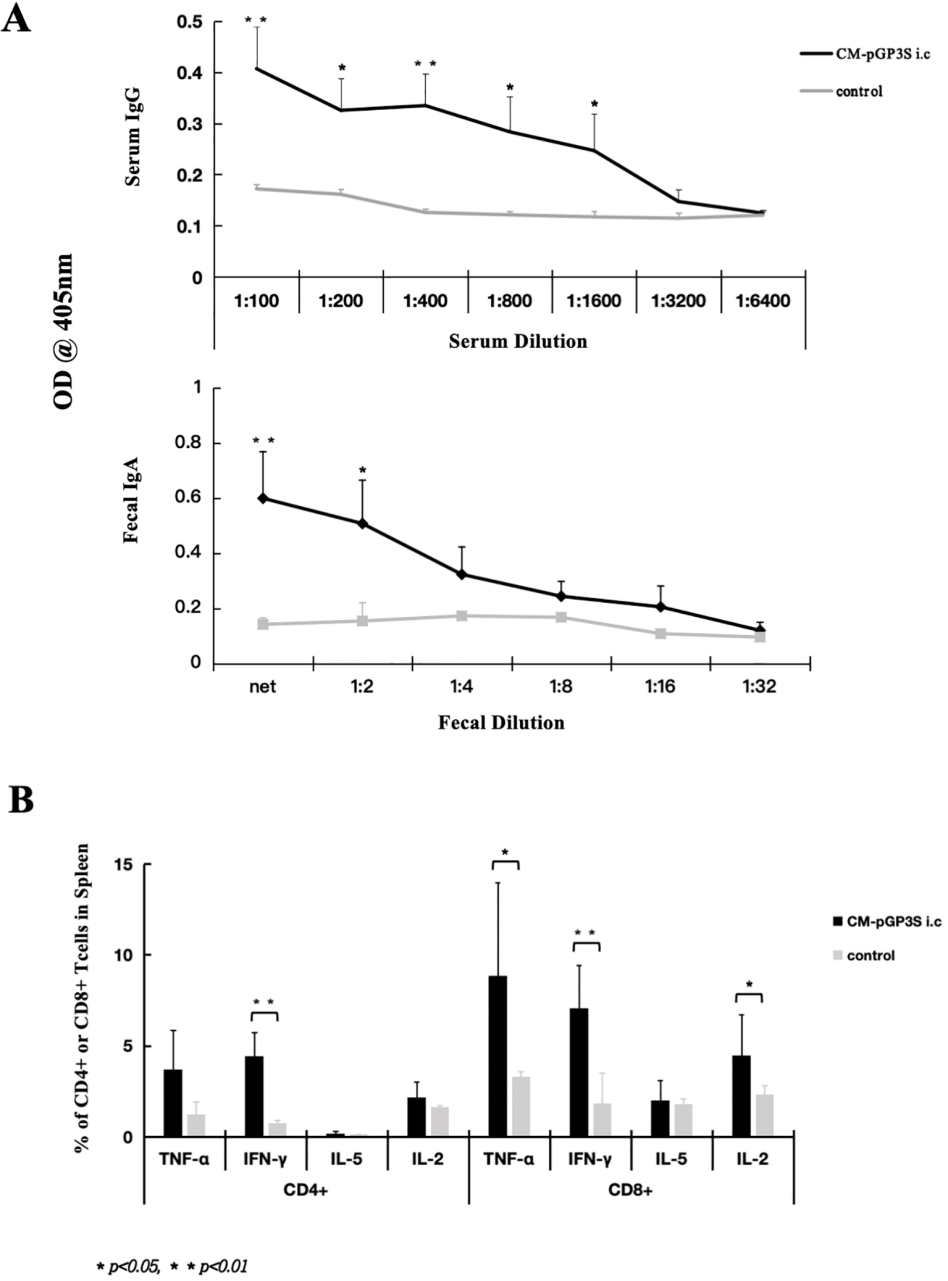
CM-pGP3S rectal immunization induces humoral and cellular immune responses. **(A)** Serum and fecal samples from mice on day 63 post-CM-pGP3S rectal immunization were tested for IgG and fecal IgA levels. The vaccine significantly increased serum IgG and intestinal IgA levels. Serum IgG levels at 1:100 and 1:400 dilutions were significantly higher than in controls (**p<0.01), with additional differences at 1:200, 1:800, and 1:1600 dilutions (*p<0.05*). Fecal IgA levels also increased compared to controls at net to 1:2 fecal dilutions (**p<0.01, *p<0.05). **(B)** Cytokine analysis of spleen CD4+ and CD8+ T cells on day 63 post-rectal immunization with CM-pGP3S showed increased IFN-γ levels in both T cell subsets, indicating a role in mucosal immunity (**p<0.01). TNF-α and IL-2 expression in CD8+ T cells was significantly higher than in controls (*p<0.05).

We used CM-pGP3S to induce transmucosal immunoprotection to elicit gastrointestinal mucosal immune responses, promoting lymphocyte proliferation. As mucosal tissue contains mucosal lymphoid tissue, which produces IgA to prevent pathogen invasion, CM-pGP3S rectal immunization increased serum-specific IgG and intestinal fecal IgA levels. Recognized epitopes were displayed on the surface of CM EBs coated on ELISA plates. IgG levels were significantly higher than in the immunized group after high-dose (1×10^7^) CM-pGP3S rectal immunization on day 63 from 1:100 to 1:1600 serum dilution, with 1:100 and 1:400 dilutions showing the highest difference (*p* < 0.01) and 1:200, 1:800, and 1:1600 dilutions also showing significant differences (**Figure 4A**, p < 0.05). Fecal IgA levels increased after high-dose (1×10^7^) CM-pGP3S rectal immunization on day 63 from neat to 1:2 fecal dilution (**Figure 4A**, p < 0.01, *p* < 0.05). Analysis of spleen cells from mice immunized with CM-pGP3S (1×10^7^) on day 63 via flow cytometry showed increased expression of cytokines by CD4+ and CD8+ T cells, particularly IFN-γ, a key player in mucosal immunity (**Figure 4B**, p < 0.01). The number of TNF-α and IL-2 expressing CD8+ T cells was also significantly higher than in the control group (**Figure 4B**, p < 0.05). These findings suggest that CM-pGP3S rectal immunization can stimulate the production of humoral and cellular immunity.

## 4 Discussion

Genital tract CT infection is a common sexually transmitted disease, often leading to hydrosalpinx and other serious complications (Hocking et al., 2023). Despite extensive efforts, no effective *Chlamydia* vaccine has been approved for clinical use (Olivares-Zavaleta et al., 2014; Qu et al., 2015; Jiang et al., 2017; Schmidt et al., 2017). Recent studies suggest that a *Chlamydia* digestive tract vaccine might be a promising approach. The presence of *Chlamydia* in the digestive tract could provide immune protection against reproductive tract infections, reducing the duration and intensity of *Chlamydia* genital tract infection and preventing complications such as oviduct hydrosalpinx (Hou et al., 2018; Wang et al., 2018). We used CM-pGP3S as a live attenuated vaccine for rectal inoculation, with protection against upper genital tract pathology assessed through infertility and tubal inflammation markers. The results indicate that rectal immunization induces cross-reactive immune responses in the lower digestive tract, reducing the length and intensity of CM-WT genital shedding and preventing CM-WT–induced hydrosalpinx. These findings highlight the potential of rectal immunization as a novel route for inducing protective immunity in the female genital tract.

In this study, a CM infection model was used to evaluate CT’s effects on genital tract infection invasiveness. Previous findings suggest that oral CM inoculation through the mucosal membrane induces resistance to subsequent CM vaginal challenges (Wang et al., 2018). Transmucosal protection is rapidly induced and can persist long-term. The vaccine inoculation route plays a crucial role in immune response induction, effectively preventing oviduct and uterine inflammation through humoral immunity. A suitable route of administration could induce immune responses in both mucosal and systemic tissues (Mayr et al., 2012). Although CM-WT colonization of the digestive tract does not cause intestinal mucosa inflammation or other pathological changes (Zhong, 2018), using the pathogenic wild-type CM-WT as a vaccine is not safe. Evidence shows that CT can directly infect the rectum, potentially causing severe proctitis or rectal colitis and increasing the risk of CT rectal transmission to the genital tract (O’Farrell et al., 2008; White, 2009). Recent studies indicate that the deficient CM-pGP3S strain does not cause hydrosalpinx or other genital tract pathologies (Hou et al., 2018). Therefore, the use of non-pathogenic attenuated CM-pGP3S as a digestive tract vaccine for intestinal mucosal immunity could enhance safety and help reduce persistent *Chlamydia* genital infections and irreversible pathologies like oviduct hydrosalpinx. Currently, an oral attenuated CM-pGP3S vaccine has been developed to induce mucosal immunity, potentially preventing genital *Chlamydia* infection and related pathogenicity (Zhou et al., 2022).

This study further explored rectal immunization with CM-pGP3S targeting the digestive tract. When CM-pGP3S was administered rectally, no pathogenicity was observed in the upper tract, indicating that CM-pGP3S is safe for the digestive system. Rectal immunization with CM-pGP3S induced robust transmucosal protection against CM-WT vaginal challenge. These findings support previous results, suggesting that the CM-pGP3S rectal vaccine provides a significant immune effect, resulting in a notable reduction in both the frequency and duration of *Chlamydia* infections following CM-WT vaginal challenge. Even at a low dose, CM-pGP3S conferred substantial protection. Rectal inoculation of wild-type CM (non-attenuated strain) in animal models can induce immune protection, but it may cause intestinal inflammation or spread to the vagina to cause reproductive tract infection (Zhong, 2018), while the attenuated strain CM-PGP3S in this study is non-pathogenic to avoid this risk. In addition, the study attempted to apply a low-dose attenuated vaccine to rectal inoculation, and the CM-pGP3S were cleared on the 63rd day after inoculation, which had no pathogenic effect on mice and could induce an immune protection effect in the vagina. After CM-WT infection, the persistent rectal infection of CM-WT disappeared on D49, thus laying the foundation for subsequent research on a low-dose attenuated vaccine.

Protective effects on the genital tract were confirmed through both macroscopic and microscopic pathological examinations. Notably, the CM-pGP3S rectal vaccine-induced protective immunity in the female genital tract, offering strong defense against *Chlamydia*-related genital diseases and presenting a novel approach to *Chlamydia* vaccine development targeting upper genital tract pathology. Despite prolonged colonization in the digestive tract, CM-pGP3S did not exhibit any significant pathology. This study thoroughly examined both lower digestive tract tissues and intestinal flora. Macroscopically, no signs of colitis were observed, and microscopically, CM-pGP3S did not induce significant inflammatory infiltration in intestinal epithelial cells nor cause major alterations in gut microbiota flora.

Using attenuated CM-pGP3S for rectal inoculation protected mice from *Chlamydia* genital infection and lesions in the upper genital tract. Rectal inoculation is appealing for immunization against genital *Chlamydia* infection as it provides transmucosal immunity through common immune effectors against mucosal pathogens such as CT. Additionally, the rectal approach avoids some adverse effects associated with traditional injectable immunizations and is considered a convenient mucosal immune strategy against microbial pathogens (Tsai et al., 2023).

This study also demonstrated that CM-pGP3S rectal immunization significantly increased serum IgG and fecal IgA, indicating strong systemic and localized intestinal antibody responses, consistent with previous research on CM-WT oral vaccines (Wang et al., 2018; Shillova et al., 2020). Although some studies have suggested that IgG antibodies may be involved in *Chlamydia* immunoprotection through neutralization or opsonophagy (Quigley and Timms, 2021), other data (Mathias et al., 2014) have not observed a significant association between IgG and infection protection. Therefore, the specific role of IgG in primary *Chlamydia* infection still needs to be further studied and may be influenced by the host immune background or antibody subtype specificity. Mucosal immunoglobulin A (IgA) has been demonstrated to perform critical immunosurveillance functions in gastrointestinal homeostasis, particularly through its regulation of commensal microbiota composition and prevention of pathogenic bacterial translocation (Crank et al., 2019).

Immunologic evaluation showed that rectal CM-pGP3S immunization induced a cellular immune response characterized by high levels of *Chlamydia*-specific CD4+ and CD8+ T cells that secreted IFN-γ, with increased TNF-α and IL-2 levels in CD8+ T cells. CD4+ T cells have been shown in both human and animal studies to be vital in CT immunity (Murthy et al., 2007). Studies have highlighted antibodies’ central role in CM-induced immunity, with effector cell activation in genital tract tissues dependent on CD4+ T cells (Farris et al., 2010). IFN-γ from CD4+ T cells activates these effector cells, promoting antibody-mediated anti-*Chlamydia* immunity (Naglak et al., 2016). CD8+ T cells, known as cytotoxic T lymphocytes, resist various pathogens, including different organisms (St Paul and Ohashi, 2020). The elevated TNF-α, IFN-γ, and IL-2 levels in CD8+ T cells observed in this study suggest that these inflammatory factors play essential roles in the immune response and defense against pathogen infections elicited by CM-pGP3S rectal immunization.

Our study demonstrated that rectal inoculation induces immune responses and mediates CM clearance in the reproductive system, indicating cooperative mucosal interactions between the rectum and reproductive tract, acting as sites for mucosal induction and effect. Within the mucosal immune system, vaccine-induced immunity has distinct functions in preventing *Chlamydia* infection sequelae, such as infertility and upper genital tract inflammation. Collectively, these results highlight rectal delivery as a viable approach for eliciting mucosal pathway-mediated protection in the female reproductive tract.

There are many studies that used live, live-attenuated, or killed *Chlamydial* EBs delivered via the intranasal route, effectively demonstrating notable protection correlates, such as heightened serum and vaginal *Chlamydia*-specific IgG/IgA, reduced time-to-clearance, reduced burdens, and subsequently reduced pathology. Indeed, intranasal EB immunization provides near complete protection, while intramuscular EB provides partial protection (Phillips et al., 2019; Zuo et al., 2021; Poston, 2024). The protective effect was found to be associated with reduced TNF-α response by antigen-specific CD8+ T cells, which mediate this inhibition. It suggests that intranasal immunity may mitigate pathology by modulating T-cell responses but requires a specific form of antigen (such as live EB) (Murthy et al., 2024). Poston et al (Poston et al., 2025). used intranasal CPAF plus ADU-S100 adjuvant to induce a CD4+ T cell response and reduce bacterial shedding and duration of infection. It suggests that intranasal immunization combined with specific adjuvants can effectively enhance mucosal immunity and shorten clearance time. Still, the present study chose to use the rectal route for several reasons. More targeted: rectal immunization more directly simulates the natural infection path and induces a local immune response in the rectal and vaginal areas (Zhu et al., 2012). In addition, the rectal area is larger than the intranasal area, allowing for more effective contact of the vaccine with the mucosa, and intranasal immunization may be more focused on local immunity of upper respiratory tract mucosa. Safety: intranasal immunization may cause local inflammation of the respiratory tract, while rectal immunization, as shown in this study, did not show significant intestinal pathology or flora disturbance in mice models. Compliance: Mucosal immunization is more acceptable to patients than intramuscular injection (Neutra and Kozlowski, 2006; Parez et al., 2006; Song et al., 2024). Furthermore, a mucosal *Chlamydia* vaccine may offer better protection at the mucosal surfaces where the infection occurs, while intramuscular vaccines focus on systemic immunity (Stary et al., 2015; de la Maza et al., 2021).

Notably, CM-pGP3S in the gastrointestinal tract induced transmucosal immunity that protected mice from subsequent genital CM-WT infection. However, CM-WT self-inoculation between the female reproductive tract and anorectum may lead to persistent CM infection in the rectal mucosa, thus prolonging the infection cycle and increasing the risk of chronic inflammation or secondary complications. Therefore, future studies should be devoted to exploring the dual effects of shortening rectal mucosal colonization time and improving reproductive tract immune protection by the development research of attenuated CM vaccines. In this study, only serum IgG, fecal IgA, and spleen CD4+ and CD8+ T cells of the high-dose immunization group (1×10^7^ IFUs) and the control group were detected on the 63rd day of immunization. WT-CT detection after vaginal infection was not performed. It will be investigated in future studies.

In conclusion, this study demonstrated that rectal immunization induces immune effectors that facilitate *Chlamydia* clearance in the genital tract, reflecting cooperation between rectal and genital mucosae as mucosal inductive and effector sites within the common mucosal immune system. Our findings underscore the potential of a rectal vaccine as an effective mucosal route for inducing protective immunity in the female genital tract.

## Data Availability

The original contributions presented in the study are included in the article/Supplementary Material. Further inquiries can be directed to the corresponding author.
